# Cryoballoon Ablation of Atrial Fibrillation Without Demonstration of Pulmonary Vein Occlusion—The Simplify Cryo Study

**DOI:** 10.3389/fcvm.2021.664538

**Published:** 2021-05-26

**Authors:** Michael Kühne, Sven Knecht, Florian Spies, Stefanie Aeschbacher, Philip Haaf, Michael Zellweger, Beat Schaer, Stefan Osswald, Christian Sticherling

**Affiliations:** Department of Cardiology, University Hospital of Basel, Basel, Switzerland

**Keywords:** atrial fibrillation, cryoballoon ablation, radiation dose, fluoroscopy, pulmonary vein isolation

## Abstract

**Background:** The demonstration of pulmonary vein (PV) occlusion is routinely performed and considered a prerequisite for successful cryoballoon (CB) ablation of atrial fibrillation (AF). The purpose of this study was to assess the feasibility and impact on procedural parameters and outcome of a standardized procedural protocol without demonstrating PV occlusion.

**Methods and Results:** Consecutive patients undergoing CB pulmonary vein isolation (PVI) were studied. After cMRI assessment, patients treated by PVI using a novel no-contrast (NC) protocol without routine contrast injections to demonstrate PV occlusion (NC group) were compared to patients undergoing PVI with contrast injections to demonstrate PV occlusion (standard group). One hundred patients with paroxysmal or persistent AF (age 61 ± 10 years, ejection fraction 59 ± 11%, left atrial volume index 37.2 ± 2.0 mL/m^2^) were studied. The NC protocol was feasible in 72 of 75 patients (96%). Total procedure time and fluoroscopy time were 64.0 ± 14.1 min and 11.0 ± 4.6 min in the NC group and 92.0 ± 25.3 min and 18.0 ± 6.0 min in the standard group, respectively (all *p* < 0.001). Dose area product was 368 ± 362 cGy^*^cm^2^ in the NC group compared to 1928 ± 1541 cGy^*^cm^2^ in the standard group (*p* < 0.001). Forty-five of 75 patients (60%) in the NC group and 16 of 25 patients (64%) in the standard group remained in stable sinus rhythm after a single PVI and a 1-year follow-up (*p* = 0.815).

**Conclusions:** Performing CB ablation without using contrast injections to demonstrate PV occlusion was feasible, resulted in reduced radiation exposure, and increased the efficiency of the procedure.

## Introduction

Pulmonary vein (PV) isolation (PVI) is the cornerstone of catheter ablation of atrial fibrillation (AF) and may be achieved by means of radiofrequency (RF) ablation or cryoballoon (CB) ablation (1–3). CB ablation has been shown to be an effective tool for PVI and the procedure has become more efficient with the advent of single-application protocols (4–6). Whereas, the use of electroanatomic mapping systems has made nearly-radiation-free or completely radiation-free and contrast-free procedures possible (7, 8), CB ablation is still routinely guided by contrast use and fluoroscopy. Although improvements in procedural workflows and learning curves have resulted in decreased procedure and fluoroscopy times (9–11), radiation doses and contrast use with CB ablation remain considerably higher compared to RF-PVI guided by a mapping system. This is mainly due to the use of fluoroscopy during contrast injections for the confirmation of PV occlusion which are routinely performed during CB ablation of AF. However, whether this really is mandatory for successful CB-PVI has not been systematically investigated.

Therefore, with the goal of further simplifying CB ablation and reducing radiation doses and contrast use, the purpose of this study was to assess the feasibility and the procedural and clinical impact of a standardized procedural protocol without contrast injections to demonstrate and confirm PV occlusion during CB ablation of AF.

## Methods

### Study Population

The subjects of this non-randomized single-center study were 100 consecutive patients from December 2017 to July 2019 with symptomatic paroxysmal or persistent AF undergoing CB ablation using the 2nd and 4th generation CB (28 mm Arctic Front Advance and Arctic Front Advance Pro, Medtronic, MN, USA). In 75 patients, CB ablation was performed using a standardized no-contrast (NC) protocol without routine cine acquisitions and without contrast injections for the confirmation of PV occlusion (NC group). In the 25 consecutive patients who had undergone CB-PVI prior to the study, CB ablation had been performed using a standard protocol with routine contrast injections for the confirmation of PV occlusion. This group served as the historical non-randomized control group (standard group).

Local ethics committee on human research approved our study, which was conducted in accordance with the Declaration of Helsinki, and written informed consent was obtained from all patients. Prior to the procedure, cMRI was performed in all patients on a 1.5T scanner (MagnetomAvanto/Espree, Siemens, Germany) equipped with phased array body coils. A respiratory- and ECG-gated three-dimensional balanced steady-state free precession sequence was acquired in axial orientation covering the whole left atrium. Segmentation and 3D reconstruction was performed using the CartoMerge software (Biosense Webster, Diamond Bar, CA, USA). In all patients, pre-procedural exclusion criteria for CB ablation based on this reconstruction was the presence of a left common PV or early branching of the right inferior PV, potentially precluding adequate tissue contact at the right inferior PV (12).

Exclusion criteria were the presence of long-standing persistent AF, a history of a previous left atrial procedure for PVI and the documentation of typical flutter requiring an additional cavotricuspid isthmus ablation. Intracardiac thrombi were ruled out by transesophageal echocardiography before the procedure.

### Cryoballoon Ablation (Standard Group)

The ablation procedure was performed under conscious sedation using midazolam, fentanyl, and propofol. All procedures were performed by the same two operators (MK, CS), both with >10 years of experience performing CB ablation. After obtaining vascular access *via* the right femoral vein using ultrasound guidance, a deflectable decapolar catheter was inserted into the coronary sinus as a reference for transseptal puncture and for pacing. This catheter was positioned in the right subclavian vein for phrenic nerve stimulation during CB isolation of the right-sided PVs. Transseptal puncture was guided using fluoroscopy and continuous pressure recordings from the transseptal needle tip. After advancing the needle tip through the inter-atrial septum, left atrial position of the needle tip was confirmed by demonstration of a left atrial pressure curve. No intracardiac or transesophageal echocardiography was used intra-procedurally. Intravenous heparin was used to keep the activated clotting time at a target of 350 s. The intracardiac electrograms and surface electrograms were displayed on an oscilloscope and recorded at a speed of 100 mm/s.

The steerable sheath (FlexCath Advance^TM^, Medtronic, MN, USA) was advanced to the left atrium after transseptal puncture and was continuously flushed with heparinized saline. The 20 mm inner lumen spiral mapping (ILSM) catheter (Achieve^TM^ and Achieve Advance^TM^, Medtronic, MN, USA) catheter was advanced deep into the PV and used for positioning the CB at the PV antrum. The ILSM catheter was then pulled back in order to visualize real-time PV potentials whenever possible. In all cases, PV potentials were recorded at the PV ostium before ablation. Regardless of whether time-to-isolation values (based on real-time signals) were available, the procedural endpoint was the elimination of all PV potentials with the ILSM catheter positioned again at the PV ostium after ablation (entrance block). Additional testing for exit block and pacing maneuvers (e.g., to distinguish far-field from near-field signals) were only used if deemed necessary by the operator.

**Figure 1:**
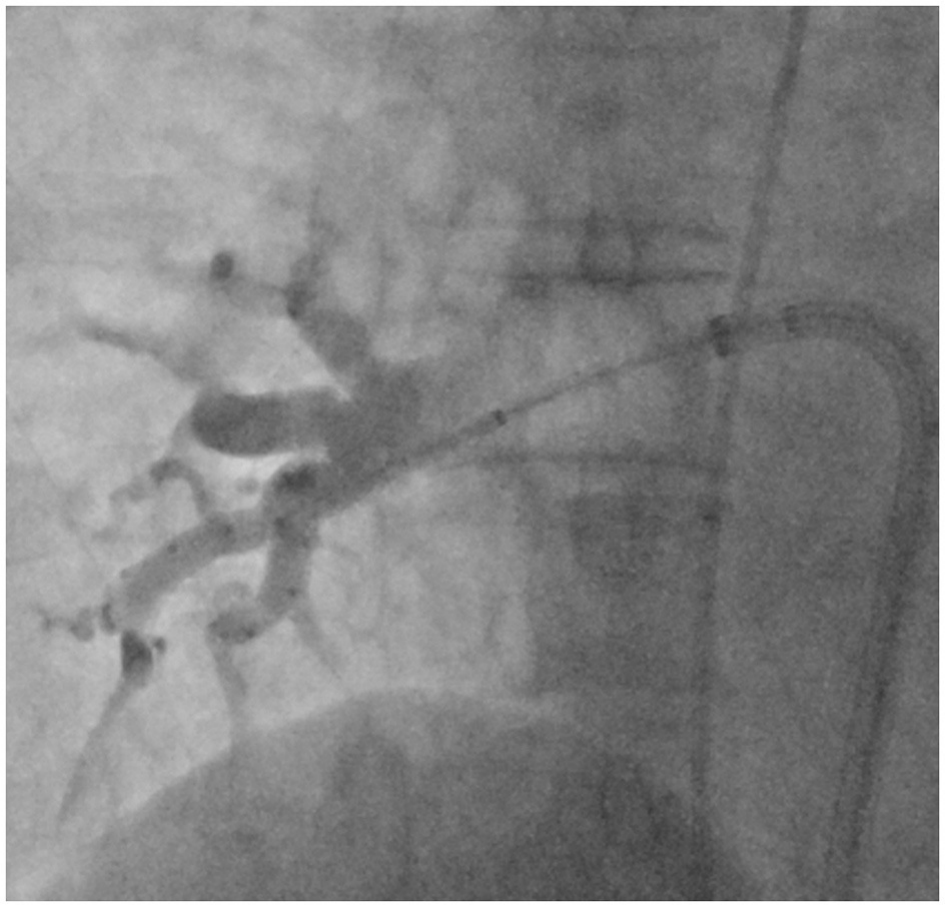
Right anterior oblique (45°) fluoroscopic view with the inflated 28 mm cryoballoon catheter positioned at the ostium of the right inferior pulmonary vein and the Achieve^TM^ inner lumen spiral mapping catheter positioned in the vein. Collimation was used to focus on the area of interest. Contrast injections to demonstrate pulmonary vein occlusion were routinely performed in the standard group, but not in the no-contrast (NC) group.

A single freezing cycle protocol with a standard duration of 180 s was started after demonstration of PV occlusion by contrast injection (Figure 1) using cine or fluoroscopy acquisition with target temperatures of −40°C and/or PV isolation (time-to-isolation) within 60 s. If this was achieved, no further catheter manipulation was performed. Pull-down maneuvers were allowed at any time but recommended if target temperatures and/or isolation were not seen within 60 s. If isolation of a PV did not occur within the first 60-90 s, termination of the freezing cycle was recommended and the catheter and/or ILSM catheter was repositioned (10, 13). If adjustment maneuvers resulted in significant additional temperature decrease within 30 s of ablation, the duration of the freezing cycle was increased to 240 s. Freezing cycles were prematurely terminated when −60°C was reached. The endpoint of the ablation was the elimination of all PV potentials on the ILSM catheter. Procedure time was measured from the puncture of the groin to the removal of the sheaths.

### Standardized Procedural Protocol (NC Group)

**Figure 2:**
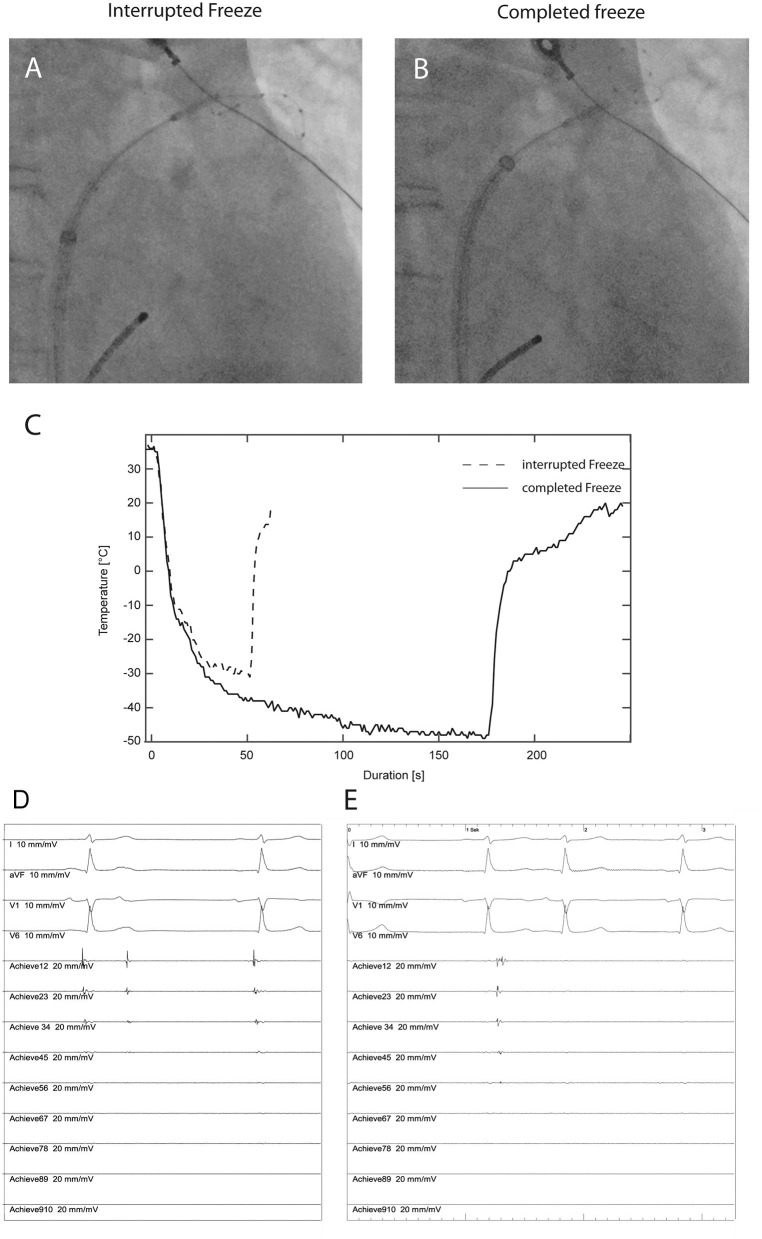
Fluoroscopic position of the cryoballoon with no contrast injection **(A)** during ablation at the superior pulmonary vein in a patient in the no-contrast (NC) group requiring interruption of the freezing cycle after 50 s due to inadequate temperature decrease (**C**, dashed line). The intracardiac recordings **(D)** from the Achieve^TM^ inner lumen spiral mapping catheter show no effect on the pulmonary vein potential (Achieve12 to Achieve45). Recordings from four surface electrocardiographic leads are also shown. After fluoroscopic adjustment of the cryoballoon position without contrast injection **(B)** an adequate temperature decrease **(C)** resulting in progressive delay and subsequent electrical isolation of the pulmonary vein **(E)**.

The following procedural aspects differed from the standard group:

Positioning of the CB was performed using tactile feedback. Right and left anterior oblique views could be used to ensure optimal CB catheter and sheath alignment with the orientation of the PV. In addition, LA anatomy was displayed (by default in right and left anterior oblique views) in addition to the fluoroscopic visualization on the additional screen in the electrophysiology laboratory during the entire procedure. No routine contrast injections and fluoroscopy acquisitions to demonstrate PV occlusion were performed (Figure 2). However, after two unsuccessful cryoapplications (not resulting in isolation or an adequate temperature decrease), contrast injections were allowed.

### Post-ablation Management

Pericardial effusion was ruled out in all patients after the procedure with transthoracic echocardiography. Oral anticoagulation was continued for at least 2 months. All antiarrhythmic drugs were stopped after the procedure. Follow-up consisted of outpatient clinic visits at 3, 6, and 12 months and included a detailed history, physical examination, 12-lead ECG, and 7-day Holter monitoring. Episodes of AF (>30 s) were counted as recurrences. Recurrence rates were analyzed with a post-procedural blanking period of 3 months. In patients with symptomatic AF recurrence, a repeat procedure was performed using focal RF ablation with a 3D electroanantomic mapping system (CARTO3, Biosense Webster). Location and type of gap was defined as focal or segmental (14). Briefly, the gap in the ablation line was defined as focal if the PV re-isolation was achieved with up to two focal applications of RF energy or segmental if >2 applications were needed to achieve PV re-isolation.

### Outcome Measures

The main outcome measures were the feasibility of the standardized procedural protocol to achieve acute pulmonary vein isolation in the NC group and the impact of the protocol on radiation dose, procedure time, fluoroscopy time, and freedom from AF or atrial tachycardia (AT) during follow-up. Secondary outcome measures were use of contrast media, total freezing time, total number of interrupted freezing cycles (due to inadequate temperature drop and/or no effect), complications, and the pattern of recurrence.

### Statistical Analysis

Continuous variables are presented as mean ± one standard deviation or as median and interquartile range (IQR) in case of skewed distribution. For continuous variables, comparisons were made using Student's *t*-test, or Mann-Whitney *U*-test, as appropriate. Discrete variables were compared using chi squared test. Analysis was performed using SPSS (IBM SPSS Statistics, Version 22.0. Armonk, USA). A *p*-value <0.05 was considered to indicate statistical significance.

## Results

### Baseline Characteristics

The study population consisted of 100 consecutive patients undergoing CB-PVI (73% male, age 61 ± 10 years). The mean left ventricular ejection fraction was 59 ± 11%, indexed left atrial volume (LAVI) was 37.2 ± 12.0 ml/m^2^. Mild to severe loss of kidney function (stage 3 to 4) was observed in 10 patients (10%). Baseline characteristics are shown in Table 1. Except for the body mass index, there were no significant differences between the standard group and the NC group with regards to baseline characteristics.

**Table 1:** Patient characteristics.

	**Standard group (*n* = 25)**	**NC group (*n* = 75)**	***P*-value**
Age (years)—mean ± SD	58 ± 10	62 ± 10	0.129
BMI (kg/m^2^)—mean ± SD	28.0 ± 4.8	25.7 ± 3.3	0.046
Men—*n* (%)	22 (88)	51 (68)	0.068
Paroxysmal AF—*n* (%)	19 (76)	46 (61)	0.230
Left atrial size (mm)—mean ± SD	42 ± 6	39 ± 8	0.103
Left atrial volume index (ml/m^2^)–mean ± SD	35 ± 9	39 ± 13	0.2870
Left ventricular ejection fraction (%)—mean ± SD	57 ± 13	60 ± 10	0.235
Reduced LVEF (<55%)—*n* (%)	6 (24)	18 (24)	1.000
Hypertension—*n* (%)	11 (44)	46 (61)	0.163
CAD—*n* (%)	0 (0)	2 (2)	1.000
eGFR (ml/min/1.73 m^2^) —mean ± SD	85.4 ± 14.7	76.5 ± 19.8	0.061
Chronic kidney disease—*n* (%)Stage			0.378
1 (normal): eGFR ≥ 90 ml/min/1.73 m^2^	10 (40%)	21 (28%)	
2 (mild): eGFR = 60–89	15 (60%)	44 (59%)	
3a (mild-moderate): eGFR = 45–59	0	5 (7%)	
3b (moderate-severe): eGFR = 30–44	0	3 (4%)	
4 (severe): eGFR <30	0	2 (3%)	

*NC, no-contrast protocol; AF, atrial fibrillation; eGFR, estimated glomerular filtration rate; IQR, interquartile range; CAD, coronary artery disease*.

### Procedural Data and Impact of the No-Contrast Protocol

The procedural endpoint of PVI was reached in all patients. In the NC group, the no-contrast protocol was feasible in 72 of 75 patients (96%). Contrast injection was required in three patients of the NC group (4%) to isolate the LSPV, the LIPV and RIPV, and the LSPV and LIPV, respectively. RF “touch-up” lesions were not needed in any of the patients.

Total procedure time was 92 ± 25 min in the standard group compared to 64 ± 14 min in the NC group (<0.001). Radiation dose was significantly lower in the NC group compared to the standard group. Dose area product was 368 ± 362 cGy^*^cm^2^ in the NC group compared to 1,928 ± 1,541 cGy^*^cm^2^ in the standard group (*p* < 0.001). Fluoroscopy time was 18.0 ± 6.0 min in the standard and 11.0 ± 4.6 min in the NC group, respectively (*p* < 0.001). Nadir temperatures were similar between the two groups, as were the mean time-to-isolation values in the different PVs (reported if time-to-isolation was available). The number of PVs with a detectable time-to-isolation was different between the standard and the NC group (70 vs. 51%, respectively, *p* = 0.001). In addition, there were numerically more patients in whom freezing cycles were aborted in the NC group (40 vs. 55%) but this was not statistically significant. The procedural data in the NC group compared to the standard group are shown in Table 2.

**Table 2:** Procedural data.

	**Standard group (*n* = 25)**	**NC group (*n* = 75)**	***P*-value**
Procedure time (min)—mean ± SD	92 ± 25	64 ± 14	<0.001
LA dwell time (min)—mean ± SD	57.0 ± 18.9	34.4 ± 10.1	<0.001
Net freezing time (s)—mean ± SD	1,567 ± 657	988 ± 303	<0.001
Radiation dose (cGy*cm^2^)–mean ± SD	1,928 ± 1,541	368 ± 362	<0.001
Air kerma (mGy)—mean ± SD	82 ± 142	32 ± 57	<0.001
Fluoroscopy time (min)—mean ± SD	18.0 ± 6.0	11.0 ± 4.6	<0.001
Use of contrast media—*n* (%)	25/25 (100%)	3/75 (4%)	<0.001
Freezes LSPV—mean ± SD	1.6 ± 0.8	1.4 ± 0.7	0.30
Freezes LIPV—mean ± SD	1.7 ± 0.9	1.4 ± 0.8	0.25
Freezes RSPV—mean ± SD	1.1 ± 0.4	1.2 ± 0.6	0.46
Freezes RIPV—mean ± SD	1.6 ± 0.9	1.2 ± 0.5	0.14
Interrupted freezes—mean ± SD	0.60 ± 0.91	1.00 ± 1.21	0.09
Number of PVs with time-to-isolation	70/100 (70%)	154/300 (51%)	0.001
Nadir temperature LSPV (°C)—mean ± SD	−50 ± 5	−49 ± 6	0.485
Nadir temperature LIPV (°C)—mean ± SD	−47 ± 8	−47 ± 7	0.765
Nadir temperature RSPV (°C)—mean ± SD	−48 ± 7	−49 ± 7	0.345
Nadir temperature RIPV (°C)—mean ± SD	−46 ± 4	−46 ± 7	0.865
Time-to-isolation LSPV (s)—mean ± SD (*n* = 71)	44 ± 18	44 ± 20	0.985
Time-to-isolation LIPV (s)—mean ± SD (*n* = 68)	59 ± 36	42 ± 29	0.017
Time-to-isolation RSPV (s)—mean ± SD (*n* = 41)	40 ± 19	35 ± 19	0.324
Time-to-isolation RIPV (s)—mean ± SD (*n* = 42)	42 ± 25	38 ± 39	0.165

*NC, no-contrast protocol; IQR, interquartile range; LSPV, left superior pulmonary vein; LIPV, left inferior pulmonary vein; RSPV, right superior pulmonary vein; RIPV, right inferior pulmonary vein; LA, left atrial*.

### Complications

In this series patients, 1 of 25 patients (4%) in the standard group and 2 of 75 patients (3%) in the NC group developed transient phrenic nerve palsy during ablation at the right superior PV using the 28 mm CB. The freezing cycle was immediately terminated when decrease of phrenic nerve capture was determined. Phrenic nerve function recovered before the end of the procedure. There were no vascular access complications requiring surgical or interventional treatment or transfusion and no tamponade, and there was no stroke or transient ischemic attack in this series.

### Follow-Up

After a follow-up of 12 months, single-procedure efficacy without antiarrhythmic drugs [sinus rhythm without evidence of recurrent AF or atrial tachycardia (AT)] was 64% (16/25) in the standard group and 60% (45/75) in the NC group (*p* = 0.815). No patients were lost to follow-up. Details for the paroxysmal and persistent AF categories are given in Table 3.

**Table 3:** Freedom from arrhythmia recurrence (single-procedure success rate).

	**Standard group *n* = 25**	**NC group *N* = 75**	***P*-value**
Total freedom from AF/AT—*n* (%)	16/25 (64%)	45/75 (60%)	0.815
Paroxysmal AF—*n* (%)	15/19 (79%)	30/46 (65%)	0.379
Persistent AF—*n* (%)	1/6 (20%)	15/29 (52%)	0.187

*AF, atrial fibrillation; AT, atrial tachycardia*.

Repeat procedures were performed in 31 of the 61 patients with AF/AT recurrence [8 of 16 patients (50%) in the standard group and 23 of 45 patients (51%) in the NC group]. Neither the number of reconnected veins [1 (IQR 1-1) vs. 1 (IQR 1-2)] nor the number of focal or segmental reconnections [3 of 8 (38%) vs. 10 of 23 (43%), respectively] was different between the groups in the standard group and the NC group.

## Discussion

The main findings of this study are: (1) A standardized procedural protocol without routine contrast injections to demonstrate PV occlusion but including cMRI information is feasible in the vast majority of patients undergoing PVI using the CB. (2) The NC-protocol is associated with a lower radiation dose, shorter fluoroscopy duration, and shorter procedure time compared a standard approach. (3) The success rates of the NC-protocol are comparable to a standard approach. (4) The complication rate was low and comparable between the two groups. (5) The approach obviates the need of contrast media in the vast majority of patients.

Hitherto, the value and need of demonstrating PV occlusion has not been questioned. In clinical routine, this is usually performed by contrast injection and cine acquisitions resulting in increased radiation doses for CB-PVI. Adapting procedural protocols by reducing the frame rate and using collimation have been shown to reduce the radiation dose (11) but still using contrast injections for the demonstration of PV occlusion. A recent study by Rubesch-Kütemeyer et al. combined the use of intracardiac echocardiography to demonstrate PV occlusion with other radioprotective measures reducing the radiation dose from 4,935 cGy^*^cm^2^ to 1,555 cGy^*^cm^2^ (15). However, the reported reduced radiation of 1,555 cGy^*^cm^2^ with the reduced radiation protocol in their study was still significantly higher compared to the reported radiation dose in our study of 351 cGy^*^cm^2^. Nevertheless, they showed that procedural protocols can result in a significant reduction of the radiation dose. Independent of procedural protocols, it has to be noted that the absolute radiation dose is also significantly dependent on the type and configuration of the angiographic system used.

Although alternative means to demonstrate PV occlusion by measuring pressures (16, 17) or using transesophageal echocardiography (18) have been reported, they are not practical and are not used in clinical routine. A study by Avitall et al. showed in canines that PV occlusion can be determined by cold saline injection but the technique has not been tested in humans (19). All these approaches have in common that testing for and demonstrating PV occlusion is considered necessary in any case before performing successful CB ablation at a given PV. Generally, experienced operators reach complete PV occlusion in up to 97% of PVs (13). However, despite a perfect occlusion, premature termination or prolongation of freezing cycles, or the need for a bonus freeze is still usually triggered by the temperature profile and/or the effect within the first minute. This is because inadequate CB temperature decreases reflect PV leakage as recently shown in an *in vitro* simulation by Ghosh et al. (20). Consequently, the fluoroscopic confirmation of PV occlusion can be considered a prerequisite to start a potentially successful cryoapplication but it is not sufficient to predict an effective lesion. The rationale of the present study was to omit this procedural step previously thought to be critical for effective CB-PVI. Our findings not only with regard to procedural parameters (e.g., nadir temperatures and “time-to-effect” measurements) but also with regard to outcomes suggest that the clinical applicability of the proposed NC-protocol is given.

Nevertheless, it is clear that adequate circumferential tissue contact is needed in order to apply successful cryoablation lesions. However, this does not mean that a technical measurement is required to confirm this. Adequate tissue contact can be achieved before starting the freezing cycle (and improved during the freezing cycle) when using sheath maneuvering techniques and the anatomical information from pre-procedural MRI. In our study, we used tactile feedback in addition to sheath alignment to position the CB at the PV ostium before starting the freezing cycle and maneuvers (pull-down) during the freezing cycle to optimize tissue contact.

Furthermore, an adequate skin incision is necessary to ensure friction-free maneuvering of the transseptal sheath and to allow tactile feedback when advancing the CB to the PV ostium or when performing adjustment maneuvers. In addition, the availability of left atrial anatomical information enables the operator to choose potential target branches for placement of the ILSM catheter in the PVs. Different balloon positioning based on PV branch selection with the guidewire has been shown to result in different nadir temperatures and this was used in this study to optimize catheter orientation (13).

## Strengths and Limitations

We believe that the proposed strategy is not suitable for operators with no or little prior experience in CB ablation. Demonstrating PV occlusion by whatever means is an adequate learning tool to gather experience with sheath and balloon maneuvering, guidewire or ILSM placement techniques and with the interpretation of temperature curves during ablation. Although the amount of contrast used for CB ablation is usually relatively low, the fact that the procedure routinely involves contrast use with a standard approach could affect the decision of whether CB ablation is a suitable option for patients with impaired renal function and AF. The current study shows that contrast-free CB ablation, eliminating the risk of development of a nephropathy in general, is feasible. It makes CB ablation a valid option also in the subgroup of patients with impaired renal function, in patients with iodine contrast allergy, and in elderly patients.

This is a relatively small non-randomized study, with the risk of confounding and selection bias, analyzing the impact of a standardized protocol on procedural parameters, safety, and mid-term outcomes. It could be hypothesized that the patients in the NC group where no PV occlusion was demonstrated before ablation may have a higher likelihood of incomplete ablation. However, the finding that nadir temperatures and “time-to-isolation” measurements were similar and success rates were comparable between the two groups support the efficacy of the novel approach. It could be argued that the decrease in procedure times, fluoroscopy time and radiation dose is at least in part due to the fact that a protocol was in place that focused on procedural workflow. However, the reduction in procedure time was mainly driven by a reduction in LA dwell time suggesting that the novel protocol reduces the time needed for CB manipulation and maneuvers in order to demonstrate PV occlusion. Nevertheless, a learning curve effect cannot be excluded despite the fact that the procedures were all performed by experienced operators. From a patient perspective, the reason why the procedure times, the radiation dose and the fluoroscopy times are decreased with the novel protocol is not important. It is the equal efficacy of the procedure and the lower radiation exposure and procedure time that matter.

## Conclusions

Performing CB ablation without using contrast injections to demonstrate PV occlusion in combination with cMRI integration is feasible in the vast majority of patients. The approach is safe, effective, and associated with a decrease in radiation exposure. It further enhances the efficiency of cryoballoon ablation by simplifying the procedural workflow without impairing efficacy.

## Data Availability Statement

The original contributions presented in the study are included in the article/supplementary material, further inquiries can be directed to the corresponding author/s.

## Ethics Statement

The studies involving human participants were reviewed and approved by Ethikkommission Nordwest- und Zentralschweiz (EKNZ). The patients/participants provided their written informed consent to participate in this study.

## Author Contributions

MK and SK contributed to conception and design of the study. MK wrote the first draft of the manuscript. SK performed the statistical analysis. All authors contributed to manuscript revision, read, and approved the submitted version.

## Conflict of Interest

MK reports personal fees from Bayer, Böhringer Ingelheim, Pfizer BMS, Daiichi Sankyo, Medtronic, Biotronik, Boston Scientific, Johnson&Johnson, Roche, grants from Bayer, grants from Pfizer, grants from Boston Scientific, grants from BMS, grants from Biotronik. The remaining authors declare that the research was conducted in the absence of any commercial or financial relationships that could be construed as a potential conflict of interest.

## Abbreviations

AF: atrial fibrillation
AT: atrial tachycardia
CAD: coronary artery disease
CB: cryoballoon
CB-PVI: cryoballoon pulmonary vein isolation
eGFR: estimated glomerular filtration rate
IQR: interquartile range
LA: left atrial
LAVI: indexed left atrial volume
LSPV: left superior pulmonary vein
LIPV: left inferior pulmonary vein
MRI: magnetic resonance imaging
NC: no contrast
PV: pulmonary vein
PVI: pulmonary vein isolation
RF: radiofrequency
RSPV: right superior pulmonary vein
RIPV: right inferior pulmonary vein.
